# Lectures on the Diseases of Infancy and Childhood

**Published:** 1849-11

**Authors:** 


*Lectures on the Diseases of Infancy and Childhood.* By Charles West, M. D. Fellow of the Royal College of Physicians; Senior Physician to the Royal Infirmary for Children, etc. etc. Philadelphia: Lea & Blanchard, 1850.

We have observed with great pleasure the increasing interest manifested by the profession during the last few years, in regard to the diseases of children, considered as a distinct subject of study. That these diseases are worthy of such consideration, both by the student and physician, will, we think, be denied by none who are acquainted with the differences of organization in early and mature life, who will reflect upon the different methods of treatment rendered necessary in the child by its peculiarities of organization, or who has been taught by experience what obstacles to a correct diagnosis meet him who has not made the diseases of early life, in some degree at least, a separate and distinct study. In view of these reasons, therefore, and for the additional one of the very heavy mortality which occurs during the first few years of life, we are greatly pleased to meet with a work like the above, coming from one who has already distinguished himself as an able and accurate observer in medicine.

We shall not attempt to present the reader with a complete analysis or labored criticism of Dr. West's book, but shall merely call attention to some of the more important points discussed by the author, and give, as we pass along, our candid and impartial opinion upon his merits as a writer, and as an observer.

The work consists of a series of lectures, the substance of a considerable part of which, we learn from the preface, was addressed to the pupils of one of the London Hospitals, in the summer of 1847. The Lectures were formerly printed in the Medical Gazette, and are now collected and published as a distinct work for the first time. It is stated in the preface that they differ but little in their present form from those which appeared in the Gazette.

Dr. West's first lecture is introductory, and is devoted to the consideration of the peculiarities of disease in children, of the necessity there is for a special study of those diseases, and of the difficulties to be met with in that study. It contains also some rules for the examination of sick children, some suggestions on note-taking, and remarks on the general plan and objects of the course. This is a well written and instructive chapter, but we regret that the author has not made two lectures rather than one upon these subjects, and thrown into the second the remarks upon the peculiarities of organization in children, which are prefixed to the history off the affections of each of the systems of the body. Under the present arrangement the student learns nothing of the special characters of the circulatory and respiratory systems in the child, as compared with the adult, until he comes to page 145, where the diseases of those systems are entered upon. This, it seems to us, is to be regretted, since it is essentially necessary to be aware of the physiological characters of those important functions in order to make a correct diagnosis of any case of disease whatever;—and yet, under the existing division of subjects, the student is carried through the whole of the diseases of the nervous system without being informed as to the normal rate and other characters of the pulse and respiration.

The following eleven lectures are occupied with a description of the diseases of the nervous system, and they are all replete with instruction and interest. We would invite particular attention to those upon congestion of the brain, upon acute and chronic hydrocephalus, and upon acute inflammation of the brain. We are glad to find that Dr. West agrees with the French observers as to the almost invariable dependence of acute hydrocephalus upon tubercular disease of the meninges. Dr. West does not follow the nomenclature now generally employed in France and in this country, which designates this disease as Tubercular Meningitis, a title which has always appeared to us both more correct and expressive than that of acute hydrocephalus. Moreover, the term Tubercular Meningitis indicates the essential lesion of the disease, and refers at once to its real nature; that of acute hydrocephalus refers to one of the effects only of the essential morbid change, one too which is not always present, and does not therefore constitute a necessary part of the disorder. That effusion is not always present in the disease, is shown by Dr. West's own statement, to the effect, that he found an appreciable quantity of fluid in the ventricles in 28 out of 30 cases, leaving us to infer that in two there was no *hydrocephalus* whatever.

Our author confirms the fact already established by the recent French observers, that simple meningitis (independent of tubercular deposit,) is very rare, compared with the tubercular form of the disorder, a fact, we may be allowed to observe, not sufficiently appreciated by the profession in this country.

The diseases of the respiratory organs are treated of in the thirteen lectures following those devoted to the nervous system, and we can truly say, that we know of no work which contains, in an equal compass, an equal amount of valuable information upon these subjects. The pulmonary affections of children, especially the tubercular, are more copiously and elaborately examined in that most valuable of all the works upon diseases of early life, the treatise of MM. Rilliet and Barthez, but the details in that work are so abundant and so minute, that few who command the language have the patience to study them as they deserve, and to such the more concise and simple style of Dr. West will prove of the greatest service.

Lectures thirteen and fourteen are particularly interesting. In the former are some valuable observations on the nature, symptoms, and treatment, of the condition of imperfect expansion of the lungs in neonati, first described by Dr. Edward Jörg, under the title of Atelectasis Pulmonum. This condition in the new born child, is, we feel sure, generally confounded in this country with cyanosis depending on cardiac malformation, and we are therefore pleased to invite the attention of American practitioners to the clear account of it given by Dr. West.

In lecture fourteen our author works out with great ability the history of a fact first discovered and established by two young countrymen of that land, to which medicine owes so many of the most valuable discoveries made in pathology and practice during the last forty years. In the Archives Génèrales de Médecine, (Nos. for January, February and March, 1844,) are three papers by MM. Legendre and Bailly, entitled Nouvelles Recherches sur quelques Maladies du poumon chez les Enfants. These gentlemen show, and we think most conclusively, that several of the pulmonic lesions observed in infants and young children, which have hitherto been ascribed to inflammation, and described under the head of pneumonia, are, in fact, due to a return of the lung to its fœtal condition, or, in other words, to collapse of the air-cells. They have shown that the alterations called splenization and carnification, and supposed to depend on pneumonia, are the results, not of inflammatory processes, but of a collapsed state of the part of the lung in which they are found. More important than this, however, is their assertion that lobular pneumonia is, in the great majority of cases, bronchitis with collapse of portions of the air-cells, the supposed hepatized lobules met with in that disease being merely lobules in a state of imperfect expansion. In some rare instances, however, a true lobular pneumonia, one consisting of disseminated and distinct patches of inflammation of the parenchyma, does occur, and to this they give the title of "*pneumonie partielle.*" For the history of these new opinions, the facts upon which they repose, and the modifications rendered necessary by them in the treatment of pulmonary disorders, we must refer the reader to the papers alluded to, and to Dr. West's excellent lecture on the subject.

We are glad to find, from the remarks upon the treatment of pneumonia, that Dr. West has been led to modify his mode of administering tartar emetic in that disease since the publication of his valuable paper on the pneumonia of children in the April number, 1843, of the British and Foreign Medical Review. In that paper he states that he employed tartar emetic "in doses of a quarter of a grain to a child of two years old, repeated every ten minutes till full vomiting is produced, and continued afterwards every two or three hours, for forty-eight, or sixty hours." In the lectures at present before us, he recommends (page 199) doses of " one-eighth of a grain every ten minutes, till vomiting is produced in the case of child of two years old, and continued every hour or two hours afterwards for a period of twenty-four or thirty-six hours." It will be seen by a comparison of the two statements, that in the more recent of Dr. West's writings the doses are reduced one-half below those in the first publication, and that the remedy is directed to be continued only half the length of time. We repeat, that it has given us great pleasure to observe this change of opinion in so accurate and able a physician as Dr. West, since it goes to confirm what we have long been convinced of, to wit, that the administration of antimony to children in the doses formerly proposed by Dr. West, and recommended also by many other writers, are often dangerous in their effects. Certain it is, that these doses have proved too large for many children who have come under our hands. We believe, indeed, that the quantities recommended by our author, in his present work, are even still too large. At least, we have found them seriously violent in their effects in many cases, and as our experience has occurred chiefly amongst the generally hearty and robust children met with in private practice, in the middle and upper ranks of society, we cannot but think that they must be still less suitable for the subjects of Dr. West's experience, as these were the children of the lower classes of the London population, of such as apply at an infirmary for out door-relief.

Dr. West does not recognize the distinction of two kinds of croup, besides that form of spasmodic disorder, now generally called laryngismus stridulus, but which Dr. West speaks of as the true spasmodic croup. He denies the propriety of making a form of croup depending on slight inflammation of the larynx, without formation of false membrane, and presenting in a very marked degree the signs of spasmodic disorder of the larynx. In this view Dr. West seems to us to be in the wrong. The descriptions given by MM. Guersent, and Rilliet and Barthez, would alone suffice to convince us of the existence of two kinds of croup, located entirely in the larynx, and totally different from laryngismus stridulus; of one in which the type of the inflammation is the simple erythematous; in which the degree of inflammation is very slight; in which the nervous symptoms greatly predominate; which is vastly more frequent than the other, constituting, in fact, nineteen-twentieths of the cases called croup by the public; which is very rarely dangerous to life, and which, according to our own experience, never runs into the membranous form; and of another, in which the type of the inflammation is the membranous; in which the nervous symptoms are present, but are accompanied by more or less violent febrile reaction, and by the signs of mechanical obstruction of the air-passages; which is rare in occurrence comparatively; which generally occurs as an epidemic affection; and which is highly dangerous to life, even under the most wisely directed and energetic plan of treatment. In addition, however, to the evidence of the French authors referred to, our own experience, during eight years past, has convinced us most positively of the existence of these two forms of laryngeal disease, and of the propriety, and even necessity, of making the distinction in practice. Dr. West ascribes all the difference between the two forms to idiosyncrasy of the patient, and says that "no advantage seems to me likely to arise from constituting a new species of croup out of a modification in its symptoms, produced by the idiosyncrasy of the patient." We believe, as already stated, that there is a very marked difference between the two, one which cannot be accounted for on the supposition of idiosyncrasy alone. We think, also, that very great advantage is to be derived from the distinction, since the physician, who is aware of the distinction, and who has learned to make the diagnosis of the two forms, will not feel himself obliged, as heretofore, to bleed, leech, calomelize and antimonialize all the children to whom he is called affected with a croupal cough, or even with a nocturnal attack of croupal suffocation, under the idea that there is but one form of croup, and that croup is always dangerous; and since he will know that the great majority of such cases recover perfectly well, if treated with a warm bath, or mustard foot-bath, and an emetic, or even with a few nauseating doses of any emetic substance.

Our author's account of hooping cough is exceedingly clear and practical, and we refer the reader to it for valuable information, in regard to the complications most to be apprehended, and as to their modes of prevention and treatment. Dr. West does not believe that we possess any remedy which will cut short an attack of the disease, and in that we entirely agree with him. We are surprised, however, to find no reference to the use of alum in hooping cough, a remedy first proposed, we believe, by Dr. Golding Bird of London, and which we have found of greater efficacy in moderating the violence and frequency of the paroxysms, than any other means.

Passing over the lectures on Phthisis and Diseases of the Heart, we come next to those upon diseases of the organs of digestion and assimilation. To the latter, ten lectures are devoted, containing concise and accurate histories of the various affections of the mouth and abdominal organs. Amongst many valuable details in these lectures, we would call attention particularly to lecture twenty-seven, in which the peculiarities of the digestive organs in infancy, the evils of artificial diet, the great importance of infants being suckled, and the best kinds of artificial nourishment, are all ably and carefully considered. The remarks upon dentition and upon the different kinds of stomatitis, are also very interesting. It is in the lectures upon intestinal diseases that our author has appeared to us most to fail. So far as his remarks go, they are, like all that he has written, sensible and useful, but he has not devoted to them an amount of space and attention commensurate with their importance, at least as they shew themselves in this country.

The lecture upon the diseases of the urinary organs, and that upon abdominal tumors and infantile syphilis, are highly creditable to Dr. West's knowledge of what others have done, and to his personal observation, but they show clearly how much remains to be learned, in regard to those affections as they occur in early life.

We shall here conclude what we have to say upon the work before us, with the remark that we were surprised to find that Dr. West had crowded into a single lecture, the histories of two such frequent and important diseases of childhood as measles and scarlet fever. This is the more surprising as he quotes at the beginning of the lecture the statement of Dr. Gregory that "in an average of five years, very nearly six per cent. of the mortality of London is due to measles and scarlatina," and that 81 per cent. of this mortality occurs under five, and 97 per cent. under ten years of age. The account of scarlatina is particularly short and imperfect: in fact, it might almost as well have been left out of the book, as the student will vainly seek in it a complete history either of the symptoms, nature, or sequelæ of the disease, or an able and satisfactory method of treatment. This seems to us the more extraordinary, as of all diseases with which we are acquainted, this has appeared to be one of those most requiring an accurate knowledge of all its forms and phases, of its present and consecutive lesions, and a ready acquaintance with whatever observation and experience have taught in regard to its treatment.

In conclusion, we shall state that, after a careful perusal of Dr. West's work, we are convinced that it is one of the best publications ever issued, upon diseases of children. Parts of it, and especially the lectures upon diseases of the respiratory organs, and some of those upon the affections of the nervous system, are deserving of the highest praise for patient research, happy descriptions of symptoms, accuracy, and plain and sensible directions for treatment. The style of the author is agreeable and pleasing, and at the same time simple and perspicuous in a very high degree. We recommend the work to our American brethren, as one which they cannot read without both pleasure and profit. J. F. M.

